# The Impact of Molecular Profile Changes and Other Histopathological Parameters on Endometrial Cancer Recurrence

**DOI:** 10.3390/cancers18152454

**Published:** 2026-07-30

**Authors:** Robert Rottscholl, Johanna Winkelmann, Konstantin Fritz, Verena Gassenmaier, Isabell Goetting, Annette Staebler, Annika Simma, Sascha Hoffmann, Birgitt Schoenfisch, Stefan Kommoss, Sara Y. Brucker, Andreas D. Hartkopf, Christina B. Walter

**Affiliations:** 1 Institute of Pathology and Neuropathology, Tuebingen University, 72076 Tuebingen, Germany; robert.rottscholl@med.uni-tuebingen.de (R.R.); verena.gassenmaier@med.uni-tuebingen.de (V.G.); isabell.goetting@med.uni-tuebingen.de (I.G.); annette.staebler@med.uni-tuebingen.de (A.S.); 2 Department of Women’s Health, Tuebingen University, 72076 Tuebingen, Germany; johanna.winkelmann@med.uni-tuebingen.de (J.W.); konstantin.fritz@med.uni-tuebingen.de (K.F.); annika.simma@med.uni-tuebingen.de (A.S.); sascha.hoffmann@med.uni-tuebingen.de (S.H.); birgitt.schoenfisch@uni-tuebingen.de (B.S.); sara.brucker@med.uni-tuebingen.de (S.Y.B.); andreas.hartkopf@med.uni-tuebingen.de (A.D.H.); 3 Department of Obstetrics and Gynecology, Diak Klinikum Landkreis Schwaebisch Hall, 74523 Schwaebisch Hall, Germany; stefan.kommoss@diak-klinikum.de

**Keywords:** endometrial cancer, molecular classification, recurrence

## Abstract

**Background/Objectives**: Numerous innovations have changed diagnosis and treatment of endometrial cancer in recent years, with molecular classification being a major factor. Limited data exist on how molecular classification changes in recurrence or metastasis and what effect this may have on therapy. This retrospective study aimed to compare the molecular classification and other histopathological parameters of the primary tumor with those of recurrence or metastasis and to discuss their implications. **Methods**: A total of 43 patients with primary endometrial cancer and histologically confirmed recurrence treated at the Tuebingen University Women’s Hospital between January 2003 and January 2017 were identified within a cohort of 964 cases. Immunohistochemistry for mismatch-repair status, estrogen receptor, p53 and L1CAM, as well as next-generation sequencing of the *POLE* genes were performed. Further histopathological and clinical data were collected and statistical analyses were performed. **Results**: No POLE-mutated patients were found. A higher proportion of high-grade tumors was observed in the recurrence (30% vs. 46%). All p53-mutated patients remained p53-mutated, while a changed molecular profile of the NSMP and MMRd subtypes was observed in nine patients (21%). L1CAM remained stable in 70% of the patients. A change in molecular profile was not associated with altered prognosis. **Conclusions**: Our study underlines the importance of additional histopathological analysis in case of recurrence or metastasis for individualized therapy planning. Further studies with larger case numbers are necessary to validate our findings.

## Simple Summary

Molecular classification has substantially changed the diagnosis and treatment of endometrial cancer in recent years. However, little is known about whether these molecular features can change between the primary tumor and a later recurrence or metastasis, and what this might mean for treatment decisions. In this retrospective study, 43 patients with endometrial cancer were examined in whom tissue from both the primary tumor and the recurrence or metastasis was available. Using immunohistochemical staining and gene sequencing, the molecular classifications of both tumor samples were determined and compared. The results showed that the molecular profile changed in about 21% of patients between the primary tumor and the recurrence, although this was not associated with a worse prognosis. These findings highlight that repeating histopathological analysis at the time of recurrence may be valuable for tailoring therapy to the individual patient. Larger studies are needed to confirm these results.

## 1. Introduction

Endometrial cancer is one of the most common gynecological cancers in women worldwide. Around 420,000 new cases were diagnosed worldwide in 2022, which corresponds to around 4.3% of all cancers in women [1]. The incidence varies geographically, with higher rates observed in developed countries. Although the disease is often diagnosed in early tumor stages, the recurrence rate is approximately 15% [2]. Surgery, radiation, chemotherapy or newer targeted therapies are the current standard options for treatment depending on the stage. In early stages, however, surgery alone may be sufficient as a treatment.

The identification of four subtypes in the Cancer Genome Atlas (TCGA) has fundamentally changed the classification of endometrial carcinoma in recent years [3]. With the assignment to specific risk groups, adjuvant therapy options can now be partially escalated or de-escalated, allowing for a more individualized approach [4]. Further new prognostic markers such as L1CAM were identified [5]. In the case of metastasis, molecular classification also provides important information for the selection of systemic therapy, including targeted therapies. In other cancer types, e.g., breast cancer, it is therefore common and recommended to perform a new histological examination to determine therapy-relevant markers if metastasis occurs. In the past, this was not the standard of care in metastatic endometrial carcinoma due to the lack of direct therapeutic relevance, except in cases of uncertain imaging findings or uncertain assignment to a primary tumor. Up till now, there are only limited data on whether endometrial cancer is a disease that changes its molecular profile during the course of the disease, and the rates of altered molecular profiles during the metastasis process vary between 3.8% and 40% [6,7,8]. Since histological confirmation can also involve risks for the patient, additional procedures such as liquid biopsy or the assessment of disseminated tumor cells in the bone marrow are also used for other entities. However, this has not yet been established for endometrial carcinoma [9,10,11].

The aim of this retrospective analysis was to compare histopathological characteristics and the molecular profile of patients with endometrial carcinoma at initial diagnosis with the profile of histologically confirmed recurrence or histologically confirmed metastasis and to consider this in a clinical context.

## 2. Materials and Methods

### 2.1. Study Population

Patients treated at the Tuebingen University Hospital (Department of Gynecology and Obstetrics) for primary endometrial cancer between January 2003 and January 2017 and with a histologically confirmed recurrence in the course of the disease, or histologically confirmed progression in primarily metastatic disease, were identified (Supplementary Figure S1). The clinical and histological data were collected from patient records. All patients were treated according to the currently valid German guidelines including surgery, radiotherapy and systemic treatments [12].

This study was approved by the ethics committee of the Medical Faculty of the Eberhard Karls University and at the University Hospital of Tuebingen (reference number 114/2024BO2) with informed consent waived due to the retrospective nature of the study. It was performed in accordance with Good Clinical Practice guidelines, national laws, and the Declaration of Helsinki.

### 2.2. Molecular Classification

This study included only patients with available molecular classification data for primary diagnosis or recurrence of endometrial cancer. For the tissue of the primary tumor of patients with a primary diagnosis from 2003 up to 2013, the prognostic significance of the molecular subgroups had been previously confirmed [13]. For the patients with a primary diagnosis from 2014 up to 2017, analysis was carried out as part of this work together with the analysis of recurrent or metastatic tissue. All methods used for the primary tumors (2003–2017) have already been described [14,15,16,17]. For molecular classification, a 0.6 mm duplicate-core tissue microarray (TMA) was generated using samples taken from representative diagnostic tissue blocks. To avoid potential misclassification arising from intratumoral heterogeneity or technical issues, any aberrant, ambiguous, or non-informative TMA staining prompted repeat staining of the respective markers on full-section slides. Patients were categorized into molecular subtypes based on the Proactive Molecular Risk Classifier for Endometrial Cancer, including *POLE*-ultramutated (*POLE*mut), mismatch repair deficient (MMRd), p53 abnormal (p53abn), or no specific molecular profile (NSMP). MMR and p53 status was evaluated using immunohistochemistry. *POLE* mutation status was analyzed using next-generation sequencing (NGS). The analysis of the recurrence tissue was performed in accordance with the following instructions on whole-slide sections.

### 2.3. Immunohistochemistry

Immunostaining was performed on an automated immunostainer (Ventana Benchmark XT, Ventana, Tucson, AZ, USA). All cases were stained for ER (rtu, Roche, Basel, Switzerland), PR (rtu, Roche, Basel, Switzerland), MLH1 (rtu; Roche, Basel, Switzerland), MSH2 (rtu; Roche, Basel, Switzerland), MSH6 (rtu; Roche, Basel, Switzerland), PMS2 (rtu; Roche, Basel, Switzerland), L1CAM (1:40; Clone 14.10; BioLegend, San Diego, CA, USA), and p53 (1:400; Novocastra, Newcastle upon Tyne, UK).

L1CAM expression was scored according to percentage of positivity in tumor cells (negative, score 0 = <10%, score 1 = 10–50%, score 2 = >50%) and tumors were determined L1CAM-positive, if ≥10% (score 1 and 2) of epithelial tumor cells showed membranous L1CAM staining.

The histological and immunohistology evaluation of the metastasis was done by two pathologists, one pathologist in advanced training and an attending gynecologic pathologist with expertise in nation-wide consultations.

### 2.4. DNA Extraction

Extraction of genomic DNA from macrodissected 5 µm paraffin sections was performed using the Maxwell^®^ RSC DNA FFPE Kit and the Maxwell^®^ RSC Instrument (Promega, Madison, WI, USA) and followed by DNA quantification with the Qubit Fluorometer employing the Qubit dsDNA HS Assay Kit (Thermo Fisher Scientific, Waltham, MA, USA), according to the manufacturer’s instructions.

### 2.5. Targeted Next-Generation Sequencing

Targeted mutation analysis was performed by next-generation sequencing (NGS; Ion GeneStudio S5 prime; Thermo Fisher Scientific, Waltham, MA, USA) using an AmpliSeq Custom Panel for hotspot regions of the *POLE* (Exon 9, 10, 11, 12, 13, 14) genes.

Amplicon library preparation and semiconductor sequencing were performed according to the manufacturer’s protocols using the Ion AmpliSeq Library Kit version 2.0, the Ion Library TaqMan Quantitation Kit on the LightCycler 480 (Roche, Basel, Switzerland), the Ion 540 Kit–Chef on the Ion Chef and the Ion 540 Chip Kit (Thermo Fisher Scientific). Output files were generated by Torrent Suite (version 5.16.1).

Ion Reporter Software (version 5.18.2.0; Thermo Fisher Scientific) was used for variant calling of nonsynonymous somatic variants compared with the human reference sequence. For variant calling, Ion Reporter Software standard settings were used and called variants were visualized using the Integrative Genomics Viewer (IGV, version 2.16.2; Broad Institute, Cambridge, MA, USA) to exclude panel-specific artifacts. Variants with allele frequency (VAF) of >2% and a coverage of at least 200× were considered as a variant. The National Center for Biotechnology Information single-nucleotide polymorphism database (dbSNP; including GnomAD, ExAC, and TOPMED) was used to exclude SNPs. The datasets generated and/or analyzed during the current study are available in the European Nucleotide Archive (ENA), Accession Number PRJEB111762.

### 2.6. Statistical Analysis

Data were analyzed using R Version 4.2.2. Continuous variables such as age are shown as median and range, otherwise numbers and percentages are shown. We estimate overall survival after recurrence (date of recurrence diagnosis) depending on several variables by Kaplan–Meier plots, and also using log-rank tests. Follow-up time was estimated by the reverse Kaplan–Meier method. In all tests, a significance level of 5% was chosen.

## 3. Results

### 3.1. Patient Characteristics at Primary Diagnosis

A total of 43 patients with a histologically confirmed recurrence of endometrial cancer and available molecular classification were included in this retrospective study. Median age at primary diagnosis was 64.9 years with a range of 46.3–83.1 years, and median BMI was 27.4 kg/m^2^ with a range of 19.3–46.8 kg/m^2^. Most patients (60%) were International Federation of Gynecology and Obstetrics (FIGO) stage I (2018) in the preoperative clinical assessment. Only a few patients had a histologically confirmed lymph node involvement (n = 6, 14%) (Table 1).

### 3.2. Primary Treatment Modalities

With regard to primary treatment, 42 patients underwent surgery, and one patient had a primary inoperable disease. Laparotomy was performed in 31% of patients (n = 13), and 69% (n = 29) underwent laparoscopic surgery. Six patients (14%) received only hysterectomy with adnexectomy, while four patients (10%) additionally received a sentinel node biopsy. The remaining patients underwent systematic pelvic (n = 12, 29%) or systematic pelvic and paraaortic lymphadenectomy (n = 20, 48%).

Following the surgical procedure, 31 patients (74%) received a recommendation for further adjuvant therapy, 10 (24%) for oncological follow-up care. In 6/31 patients, the recommended adjuvant therapy was not carried out or was refused.

### 3.3. Histological and Molecular Profile of Primary Tumors

**Figure 1:**
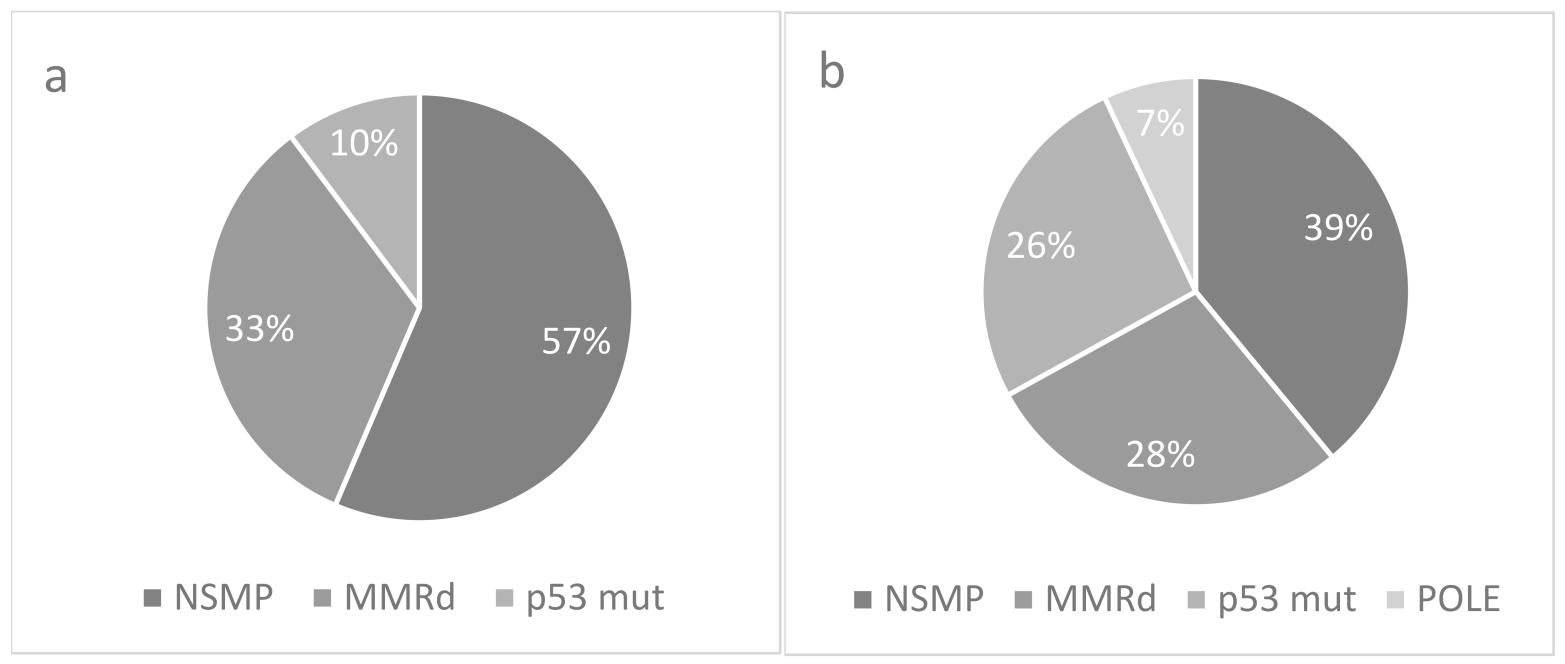
(**a**) Molecular classification of the primary tumors (n = 39); no patients with *POLE* mutation. (**b**) Molecular classification of the study cohort of the Cancer Genome Atlas Network 2013 (n = 373), modified according to [3].

Among the histological subtypes of the primary tumors, 90% of them are endometrioid carcinomas. The molecular classification resulted in 57% NSMP, 33% MMRd and 10% p53-mutated. There were no *POLE*-mutated tumors in the collected results (Figure 1).

### 3.4. Risk Stratification According to ESMO Guidelines

When considering the risk groups relevant for treatment planning according to ESMO, there are relevant changes both in the preoperative assessment (according to ESMO 2016) and postoperatively (according to ESMO 2016) as well when considering the molecular classification (according to ESMO 2021) (Table 2 and Table 3). The addition of definitive histology after surgery alone led to a higher risk grouping in 6 of 43 patients (14%) in this cohort (Table 2).

Looking specifically at the changes in the risk group postoperatively (ESMO 2016), when the molecular classification was considered, 30/43 patients (70%) showed a constant allocation. Two patients (5%) were classified higher, but seven patients (16%) were also downgraded in their risk profile (Table 3).

### 3.5. Time to Recurrence

The time to recurrence of the disease in the patients studied varied between 0.2 and 11.5 years, and the median time to relapse was 2.5 years. Peak of recurrence was in the first 2 years after primary diagnosis.

### 3.6. Comparison of Primary Tumor and Recurrence

**Figure 2:**
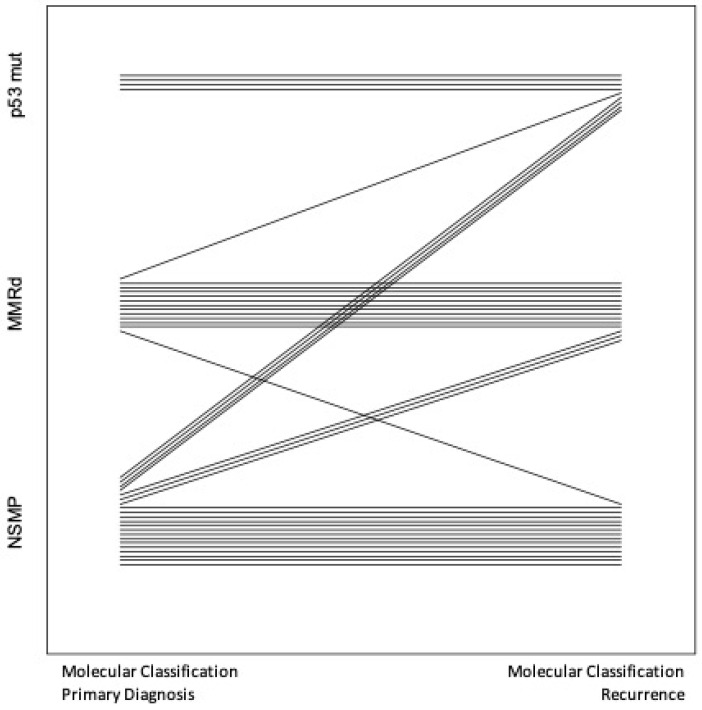
Comparison of molecular classification: primary diagnosis versus recurrence.

There was no relevant change in the histological subtype between the tissue of the primary tumor and the tissue of the recurrence (Table 4). Only two patients switched from a non-endometrioid subtype to endometrioid in the recurrence situation. Similarly, three patients with primary endometrioid carcinoma were assigned to a non-endometrioid subtype during recurrence. As expected, more high-grade tumors were found in the recurrence group. For both the molecular subtype and the estrogen receptor, a shift towards more aggressive tumor types can be seen. There was no change in the expression of L1CAM in 30 patients (70%). However, there was a change in L1CAM status in 11 patients, whereby an increase or decrease was observed in nearly equal measures. With respect to the specific pathways of change in the molecular subtype, there are no shifts in the primarily p53-mutated tumors, while the other two groups, NSMP and MMRd, were able to develop into all other groups (Figure 2). Overall, 9 out of 43 patients (21%) experienced a change in their molecular profile in the context of recurrence.

### 3.7. Survival Analysis

**Figure 3:**
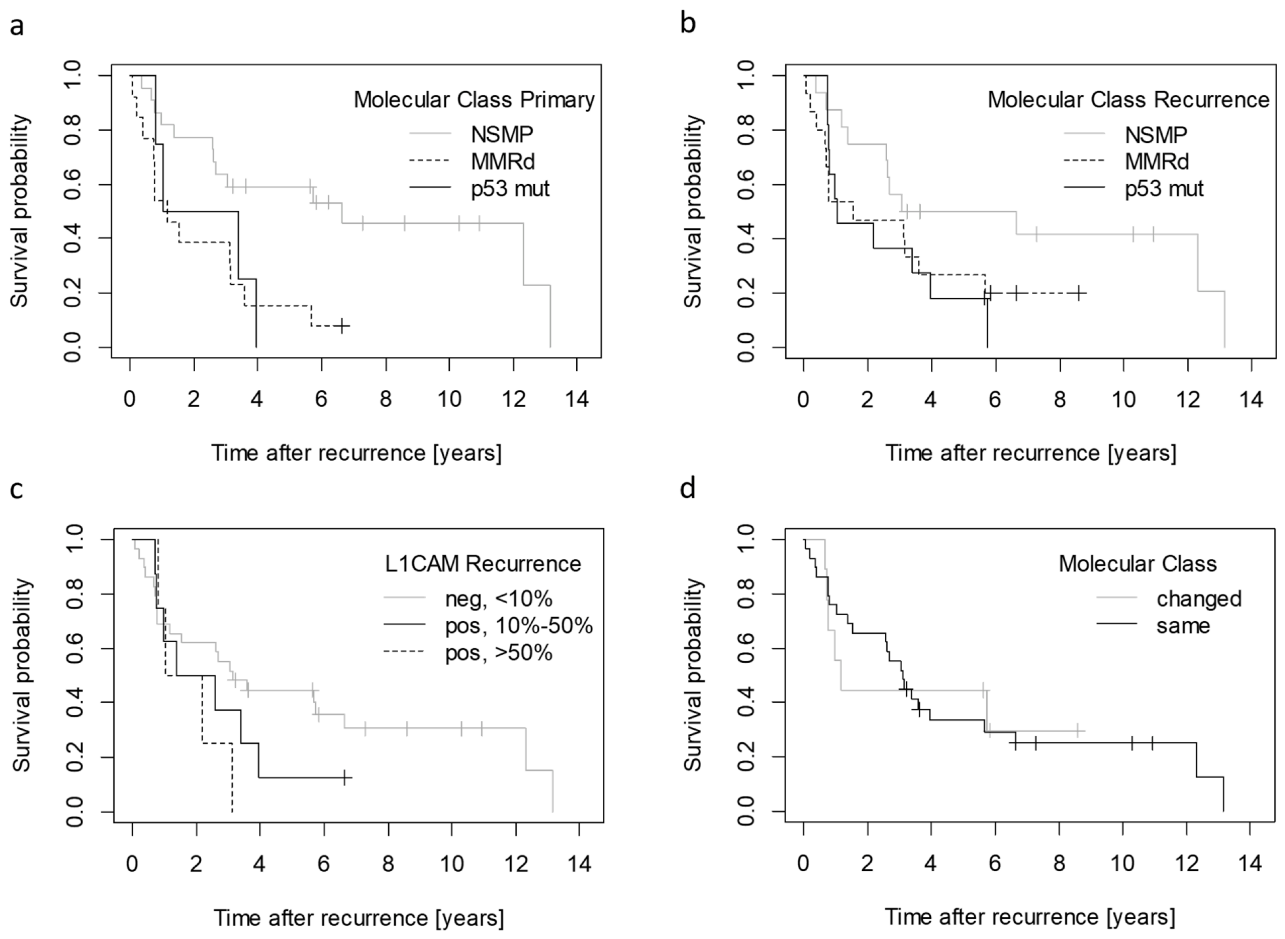
Survival analysis (overall survival): (**a**) Kaplan–Meier survival analyses according to molecular subgroups of primary tumor. (**b**) Kaplan–Meier survival analyses according to molecular subgroups of recurrence. (**c**) Kaplan–Meier survival analyses according to L1CAM status in the recurrent setting. (**d**) Kaplan–Meier survival analyses with and without change in the molecular classification between primary tumor and recurrence.

The median follow-up time after recurrence was 7.3 years, with 33 of 43 patients reported to have died during the study period. The median survival after diagnosis of the recurrence was 2.7 years with a 95% confidence interval of [1.2;5.7] years. There was a significant difference (*p* = 0.009) between the survival curves with regard to the molecular classification of the primaries of the patients, with a primary NSMP tumor showing the best survival (Figure 3a). No significant difference was found between the survival of the patients with regard to the molecular classification of the recurrent tissue (Figure 3b). Similarly, the level of L1-CAM expression in recurrent tissue did not show a statistically significant impact on overall survival (Figure 3c). An unchanged molecular status in the primary tumor compared to the recurrent tissue also showed no significant survival benefit compared to the patients who had a molecular switch (Figure 3d).

## 4. Discussion

In 2022, endometrial cancer ranked 15th among the incidence of oncological diseases worldwide in the Global Cancer Statistics. However, the incidence is expected to rise in the future [18]. In this context, longer life expectancy and lifestyle-associated risk factors—in particular obesity—play a major role [19,20]. Against this background of a rising burden, endometrial carcinoma has seen more diagnostic and therapeutic innovation in recent years than any other gynecological malignancy. The discovery of molecular subtypes in particular has led to a complete rethinking of diagnostic and therapeutic approaches [3,14]. Histopathological markers such as lymphovascular space invasion (LVSI) and L1CAM also contribute to risk assessment as prognostic markers [21,22]. Together with the classic pathological parameters such as tumor extent, grading and lymph node status, risk classifications such as those of the ESMO-ESGO et al. facilitate the right treatment decisions in adjuvant therapy [4,23]. The International Federation of Gynecology and Obstetrics also included this in its cancer staging system, leading to a new FIGO staging for endometrial cancer published in 2023 [24] with corresponding new guidelines [25].

In the metastatic and recurrent setting, the prognosis is poor and the therapeutic options are limited. In addition to classical platinum-based chemotherapy regimens, immune checkpoint inhibitors and PARP inhibitors are now used as standard options [26,27,28] as well as tyrosine kinase inhibitor combinations [29]. In contrast to other entities, histologic confirmation of metastasis was rarely performed in the past. But the efficacy of immune checkpoint inhibitors is directly linked to the MMR status of the tumor, making molecular characterization of recurrent disease particularly relevant. It is this translational question—what molecular and histopathological changes occur during recurrence, and what are their clinical consequences?—that motivates the present study.

The primary tumor characteristics of our patient cohort (n = 43 patients with histologically confirmed recurrence, diagnosed between 2003 and 2017) are consistent with the well-established clinical profile of endometrial carcinoma (Table 1 and Table 4): 90% endometrioid histology, predominantly FIGO I/II at diagnosis, and only 14% lymph node involvement. Notably, 77% of patients underwent systematic pelvic or pelvic and paraaortic lymphadenectomy, reflecting the standard of care in the time period of data collection [30,31].

The molecular distribution of the primary tumors in our cohort—57% NSMP, 33% MMRd, and 10% p53-mutated (Figure 1)—is broadly comparable to the original Cancer Genome Atlas (TCGA) cohort [3]. This also applies to the distribution of molecular subtypes in the overall cohort on which our study is based (Supplementary Figure S2). An important observation is that no POLE-mutated tumors were found in our study cohort. This underlines the assumption of an excellent prognosis of this subgroup with a low number of recurrence and the consequence of therapeutic de-escalation [32]. What is also notable is the lower proportion of p53-mutated patients.

If we now look at the ESMO risk group distribution at the time of primary diagnosis, and with the knowledge that all patients will develop a recurrence in the course of the disease, a large number of patients are still in the low-risk group, even when the molecular classification is taken into consideration (n = 11, Table 3). This supports the hypothesis that the NSMP subgroup is molecularly and clinically heterogeneous, and that further sub-classification—for example, based on estrogen receptor (ER) status and grading—may be needed to refine prognostication [33]. A limitation worth noting is that estrogen receptor (ER) status in the primary tumors was not routinely scored as part of standard clinical practice. Nevertheless, based on the primary tissue, 4 out of 22 patients with NSMP tumors could be classified into the “high-risk” NSMP group. When considering the recurrent tissue, this is the case for 3 out of 16 patients (Supplementary Figures S3 and S4).

A central finding of our study is the persistence of most histopathological markers across the primary-to-recurrence transition. The histological subtype remained concordant in the vast majority of cases (74.4% stable subtype). The ER status (in 90%) and L1CAM (in 73%) also represented stable factors in most cases in our study, even across the metastatic process. Approximately 17% of the patients showed a shift in grading (from low grade to high grade), consistent with the biological concept of clonal selection during tumor progression. The shift towards more aggressive tumor types observed in the molecular subtype data—in particular the increase in p53-mutated tumors in the recurrent cohort (from 10% to 26%)—parallels this grading shift and reflects the selective advantage of more aggressive clones during metastasis. These changes may partly be explained by processes of epithelial–mesenchymal transition (EMT), which have been implicated in the acquisition of mesenchymal and invasive properties by tumor cells during progression [34].

The molecular classification at recurrence was the primary focus of our analysis. Overall, 9 out of 43 patients (21%) experienced a change in their molecular profile at recurrence.

First, p53-mutated tumors showed complete molecular stability: in all four patients with a p53-mutated primary tumor, the p53-mutated subtype was maintained at recurrence. This finding is consistent with the notion that p53 mutation represents a fundamental driver event of high-grade endometrial carcinoma, conferring a distinctive and stable oncogenic phenotype.

Second, both the NSMP and MMRd subgroups showed the capacity to shift towards other molecular subtypes during recurrence. Importantly, no de-escalation in the form of a newly acquired *POLE* mutation was observed—which is expected, as *POLE* mutations represent early somatic events and cannot be acquired during tumor progression.

The changes shown in the molecular profile of the metastases once again reflect the heterogeneity of the NSMP group. Recent work by Timmermans et al. [33] has provided important characterization of high-grade NSMP carcinomas stratified by ER status and grading, offering a more nuanced framework for this heterogeneous group, with implications for both prognostication and treatment selection in the recurrent setting. These new data have to be validated in the context of known processes during metastasis, e.g., epithelial–mesenchymal transition (EMT) [34].

Our finding of a 21% overall rate of molecular profile change is broadly in line with the range reported in the existing literature (3.8–40%) [6,7,8]. A particularly clinically relevant mechanism of molecular profile change was recently described by Moreno-Moreno et al. [7] in a study specifically analyzing histological and molecular type changes in endometrial cancer recurrences using next-generation sequencing and MLH1 promoter methylation analysis. This study reported that in a cohort of 15 low-grade endometrioid endometrial carcinomas (LG-EEC), 13% underwent a shift from NSMP to MMRd subtype at recurrence, driven by acquired MLH1 promoter hypermethylation. This epigenetic mechanism—not detectable by standard IHC alone—represents an important route by which tumors that were initially immunotherapy-ineligible may become potential candidates for checkpoint inhibitor therapy at recurrence.

This finding from Moreno-Moreno et al. [7] is of direct clinical relevance in the context of our data. In our cohort, changes within the NSMP group towards MMRd were also observed. However, since our study predated routine MLH1 methylation analysis, the underlying epigenetic mechanisms could not be systematically evaluated. In light of the Moreno-Moreno findings, it is plausible that a proportion of the NSMP-to-MMRd shifts in our dataset were driven by the same MLH1 promoter hypermethylation mechanism. This has immediate therapeutic implications: patients with an initially NSMP-classified primary tumor should have their recurrent tissue re-tested for MMR status, ideally including MLH1 methylation analysis, as the acquisition of MMRd at recurrence would qualify them for treatment with immune checkpoint inhibitors such as dostarlimab or pembrolizumab [26,28].

McHenry et al. [8], studying recurrent and metastatic endometrioid endometrial carcinomas, and Loukovaara et al. [6], in a large cohort study of relapsed endometrial carcinoma, both reported that molecular classification retains its prognostic relevance in the recurrent setting, with NSMP tumors showing more favorable outcomes than MMRd or p53-mutated tumors. Interestingly, Loukovaara et al. [6] also observed that the pattern of relapse differs by subtype—local relapses in NSMP, regional involvement in MMRd, and distant metastases in p53-mutated tumors—a finding that has potential implications for surveillance and treatment strategies, though our cohort was too small to address this question specifically.

The poor prognosis in the event of metastasis was also confirmed in our data (median overall survival: 2.7 years, 95% CI: 1.2–5.7 years). The known prognostic significance of the primary molecular subtype [13] could be reproduced in our data based on the time of metastasis diagnosis (Figure 3a). When looking at survival with regard to the molecular subtype of the recurrent tissue, the same tendency can be seen as in the primary determination, but this data was not significant (Figure 3b) and would have to be revalidated in a larger collective. This also applies to the determination of L1CAM in the tissue of the recurrent disease (Figure 3c). Crucially, a change in molecular subtype between primary tumor and recurrence was not associated with a significant change in overall survival (Figure 3d).

Despite the lack of possible conclusions regarding the further prognosis, it was shown that certain parameters and thus also the molecular subtype can change in the course of the metastatic process. This is crucial with regard to the planning of the further therapeutic procedure and the selection of targeted and individualized therapy concepts. Determining the parameters of circulating tumor cells could represent a less invasive form of diagnostics for the future [35].

Several limitations of our study must be acknowledged. The retrospective single-center design and the limited sample size (n = 43) restrict both statistical power and generalizability. The study population was identified from a cohort treated between 2003 and 2017, a period that predates routine molecular classification and some current standard therapies. With regard to the molecular profile, no second opinion was obtained from an independent reference center. Similarly, the effect of adjuvant therapies on molecular changes could not be assessed in this analysis.

## 5. Conclusions

In summary, our data showed that the molecular profile and histopathological markers such as grading can change during the course of metastasis, while the expression of ER status and the prognostic marker L1CAM tend to remain constant. This is particularly true for the molecular subtype of NSMP carcinomas, whereas the p53-mutated carcinomas did not change their molecular subtype at all in our study. The results support the necessity of a renewed determination of specific markers for individual therapy planning in the metastatic setting. A prognostic value could not be shown here, and would have to be revalidated in a larger collective.

## Figures and Tables

**Table 1:** Clinical factors at primary diagnosis of endometrial cancer.

	Number	Percentage
All patients	43	
**International Federation of Gynecology and Obstetrics stage 2018 (n = 40)**		
I	24	60%
II	13	32%
IVB	3	8%
**Local tumor extension (n = 40)**		
pT1a	16	40%
pT1b	13	32%
pT2	4	10%
pT3a	5	12%
pT3b	2	5%
**Nodal status (n = 42)**		
Negative	36	86%
Positive	6	14%
**Grading (n = 43)**		
Grade 1	14	33%
Grade 2	16	37%
Grade 3	13	30%
**Vascular invasion (n = 38)**		
V0	33	87%
V1	5	13%
**Lymphangiosis (n = 38)**		
L0	25	66%
L1	13	34%

**Table 2:** ESMO 2016, risk profile pre- and postoperative.

Number of Patients		Risk Profile (ESMO 2016) Postoperative				
		Low	Intermediate	High-Intermediate	High	n.a.
**Risk profile** **(ESMO 2016)** **preoperative**	**low**	10	4	1	1	-
	**intermediate**	-	-	-	-	-
	**high-intermediate**	-	-	-	4	-
	**high**	3	1	-	16	-
	**n.a.**	-	-	-	-	3

**Table 3:** ESMO 2016 postoperative versus ESMO 2021.

Number of Patients		Risk Profile (ESMO 2021)				
		Low	Intermediate	High-Intermediate	High	n.a.
**Risk profile** **(ESMO 2016)**	**low**	11	1	-	1	-
	**intermediate**	-	5	-	-	-
	**high-intermediate**	-	-	-	-	1
	**high**	-	-	7	14	-
	**n.a.**	-	-	-	-	3

**Table 4:** Histology and molecular factors at primary diagnosis and at recurrence.

	Primary Tumor Number	Percentage	Recurrence Number	Percentage
**Histological Subtype**	(n = 39)		(n = 41)	
Endometrioid	35	90%	37	90%
Adenosquamous	1	3%	1	2%
Mixed	1	3%	0	0%
Muzinous	1	3%	1	2%
Serous	1	3%	1	2%
Dedifferentiated	0	0%	1	2%
**Grading**	(n = 43)		(n = 41)	
Low Grade	30	70%	22	53%
High Grade	13	30%	19	46%
**Molecular Subtype**	(n = 39)		(n = 42)	
NSMP	22	56%	16	38%
MMRd	13	33%	15	36%
P53 mut	4	10%	11	26%
*POLE*	0	0%	0	0%
**L1-CAM**	(n = 43)		(n = 41)	
Negative (<10%)	30	70%	29	71%
Positive (>10%)	8	19%	8	20%
Positive (>50%)	5	12%	4	10%
**Estrogen Receptor**	(n = 33)		(n = 41)	
<1%	4	12%	9	22%
Weak (1–50%)	7	21%	4	10%
Strong (>50%)	22	67%	28	68%

## Data Availability

The data presented in this study are available on request from the corresponding author (due to privacy and ethical restrictions).

## Supplementary Materials

The following supporting information can be downloaded at https://www.mdpi.com/article/10.3390/cancers18152454/s1. Figure S1. Flow chart of patient selection; Figure S2. Distribution of molecular subtypes in the overall cohort (n = 603); Figure S3. Classification of NSMP subgroups of the primary tumor; Figure S4. Classification of NSMP subgroups of the recurrence.

## Abbreviations

The following abbreviations are used in this manuscript:

BMI	Body Mass Index
EMT	Epithelial–Mesenchymal Transition
ER	Estrogen Receptor
ESMO	European Society For Medical Oncology
ESGO	European Society of Gynaecological Oncology
FIGO	International Federation of Gynecology and Obstetrics
IHC	Immunohistochemistry
L1CAM	L1 Cell Adhesion Molecule
LG-EEC	Low Grade Endometrioid Endometrial Cancer
LVSI	Lymphovascular Space Invasion
MMR	DNA Mismatch Repair
NSMP	No Special Molecular Profile
PARP	Poly (ADP-Ribose) Polymerase
POLE	DNA Polymerase Epsilon
TCGA	The Cancer Genome Atlas
