# Supplementary material for: The Impact of Molecular Profile Changes and Other Histopathological Parameters on Endometrial Cancer Recurrence
Source: Cancers (Basel). 2026 Jul 30;18(15):2454. doi: 10.3390/cancers18152454 (PMC13464895; doi:10.3390/cancers18152454)
Supplement: Supplementary file 1 [file cancers-18-02454-s001.zip › cancers-4417859-supplementary.pdf]

SUPPLEMENTARY INFORMATION

Supplement Figure S1

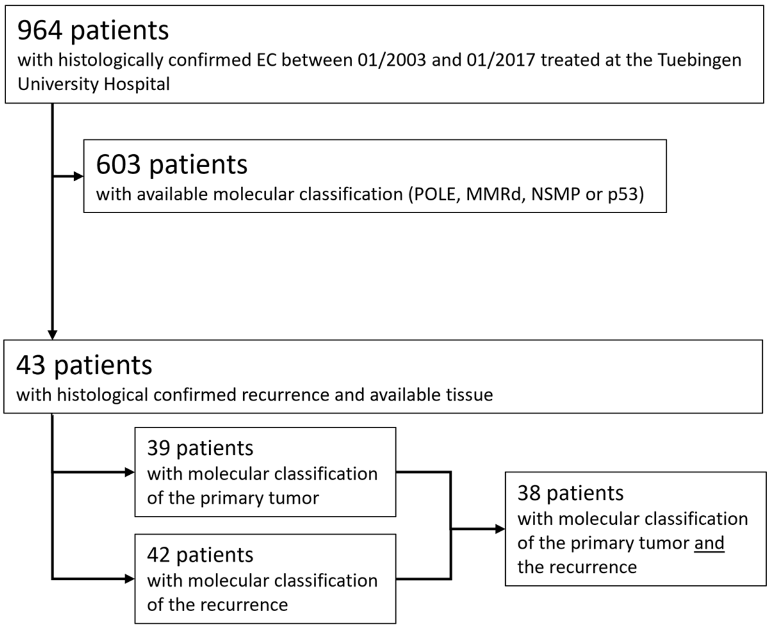

Supplement Figure S1. Flow chart of patient selection

Supplement Figure S2

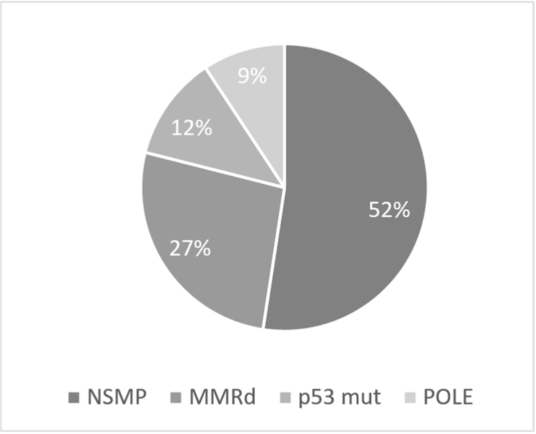

Supplement Figure S2. Distribution of molecular subtypes in the overall cohort (n=603)

Supplement Figure S3

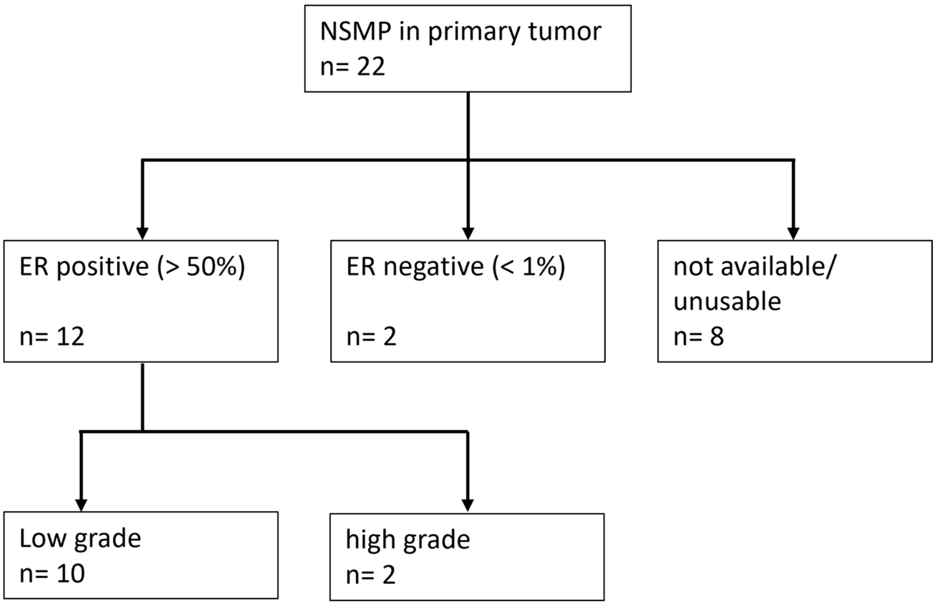

Supplement Figure S3. Classification of NSMP subgroups of the primary tumor

Supplement Figure S4

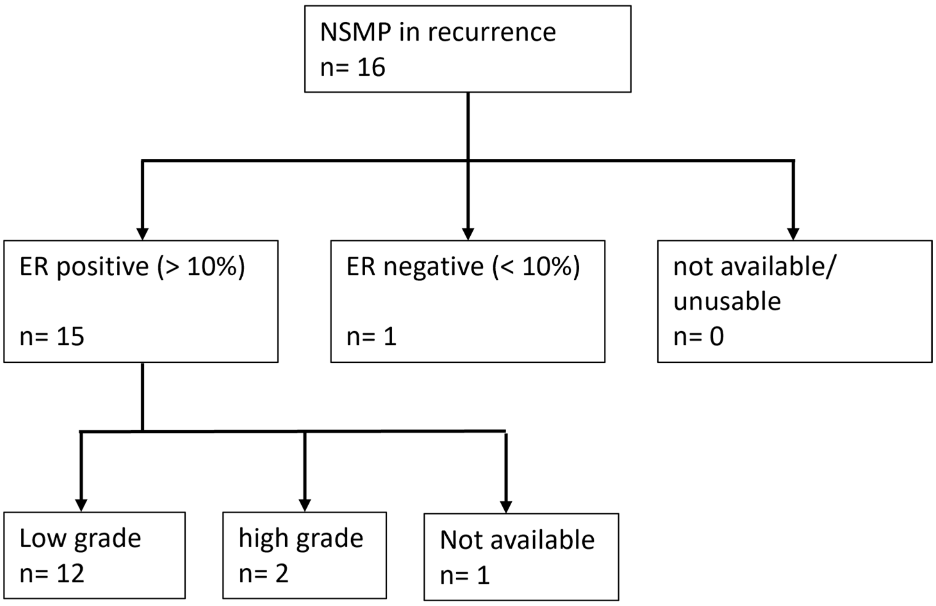

Supplement Figure S4. Classification of NSMP subgroups of the recurrence
